# Interleukin-15 and Tumor Necrosis Factor-*α* in Iraqi Patients with Alopecia Areata

**DOI:** 10.1155/2023/5109772

**Published:** 2023-05-10

**Authors:** Zainab A. Kamil, Galawish A. Abdullah, Haider Hashim Zalzala

**Affiliations:** ^1^Ministry of Health, Baghdad, Iraq; ^2^Department of Medicine, Al-Kindy College of Medicine, University of Baghdad, Baghdad, Iraq; ^3^Head of Microbiology Department, Al-Kindy College of Medicine, University of Baghdad, Baghdad, Iraq

## Abstract

**Background:**

Alopecia areata (AA) is a common form of noncicatricial hair loss of unknown cause, affecting 0.1-0.2% of the general population. Most evidence supports the hypothesis that it is disease of the hair follicle of autoimmune nature mediated by T-cells, with important cytokine role. *Objective of the Study*. The objective of this study is to study the association and changes in serum levels of interleukin-15 (IL-15) and tumor necrosis factor-*α* (TNF-*α*) in patients with AA in relation to the type, activity, and disease duration. *Patients and Methods*. Thirty-eight patients with AA and 22 individuals without the disease as controls were enrolled in this case-controlled study conducted in the Department of Dermatology in the Al-Kindy Teaching Hospital and Baghdad Medical City, Iraq, during a period from the 1st of April 2021 to the 1st of December 2021. Serum concentrations of IL-15 and TNF-*α* assessed using the enzyme-linked immunosorbent assay.

**Results:**

The mean serum concentration values for IL-15 and TNF-*α* were higher significantly in patients with AA than in controls (2.35 versus 0.35 pg/mL and 50.11 versus 20.92 pg/mL, respectively). IL-15 and TNF-*α* showed no statistically significant differences in level in terms of the type, duration, and activity of the disease, but TNF-*α* significantly higher in those with totalis-type than in other types.

**Conclusion:**

Both IL-15 and TNF-*α* are markers for alopecia areata. The level for these biomarkers was not affected by duration or disease activity, but it was affected by the type of disease, as the concentrations of IL-15 and TNF-*α* were higher in patient with Alopecia totalis than in other types of Alopecia.

## 1. Introduction

Alopecia areata (AA) is a common inflammatory noncicatricial type of hair loss with an unexpected course and a wide spectrum of clinical manifestations. While males and females have the same chance of affection, some data have revealed that men have the chance for an earlier diagnosis in comparison to females that present in adolescence with the association of involvement of nails and autoimmune diseases. The prevalence of disease ranges from 0.1% to 0.2% worldwide. The exact aetiopathogenesis is unknown, but there are multiple hypotheses, including genetic, environmental, and autoimmune pathogenesis [1, 2].

The autoimmune pathogenesis is either through destruction of the hair follicle by inflammatory cells, in particular cytotoxic T-cells through the production of gamma interferon, which activates interleukin 2, 7, 15, and 21, and these cytokines signal the Janus kinase signal transducer and activator of transcription (JAK/STAT) pathway or through loss of the hair follicle's immune privilege, leading to its destruction by the immune system [3].

Interleukin (IL)-15 is an inflammatory cytokine that has multiple effects on different cell types. Both innate and acquired immune systems can be affected by IL-15, explaining its participation in inflammation and immune response to infection [4]. IL-15 acts through the JAK-1 and JAK-3 pathways [5]. In AA both IL-15 and its IL-15R*β* receptor subunit levels are elevated in the affected hair follicles [6].

The tumor necrosis factor alpha (TNF-*α*) is an inflammatory cytokine that is involved in AA pathogenesis and several autoimmune inflammatory disorders like psoriasis and systemic lupus erythematosus, for which the level of this cytokine is found to be relevant with the disease severity and activity, while in AA, only a few studies have measured the level of TNF-*α*, and the results were controversial [7].

## 2. Patients and Methods

This case-controlled study was performed in Dermatology unit in the Al-Kindy Teaching Hospital and Dermatology Center of Baghdad Medical City, Iraq, from the 1st of April 2021 to the 1st of December 2021, in line with the guidelines of the Helsinki Declaration and items Strengthening the Reporting of Observational Studies in Epidemics (STROBE) statement.

The study involved thirty-eight (38) patients with alopecia areata of any age, gender, and type who attended the outpatient clinics. The control group consisted of twenty-two (22) generally healthy people.

Exclusion criteria:

1. Patient with any type of alopecia areata on treatment.
2. Any patient with associated inflammatory, infectious, malignant, and autoimmune diseases affecting the skin or other systems of the body in addition to alopecia areata.
3. COVID-19 patients.
4. Immunosuppressed patients or those on immunosuppressive therapy.
5. Pregnant and lactating women.

A directed interview was done to get a complete history from the patients including age, gender, time of onset for AA, previous history of the same condition and the time of it, medical history, and history of AA or autoimmune disease in the patient's family.

The scalp examination was done for evaluating the sites and number of patches of AA, detecting exclamation mark hairs presence, and for the pull test. The body's hairy area was examined meticulously for alopecia patches. Nail involvement was detected through careful nail examination.

Patients with AA were divided clinically into four groups: (I) alopecia unilocularis, (II) alopecia multilocularis, (III) alopecia totalis, and (IV) alopecia universalis [8].

For the disease duration, three groups of patients were included (I) Six months or less, (II) >six months but <1 year, and (III) one year or more [8].

Venous blood was collected in a gel tube by venipuncture of five milliliters from both patients and the healthy person under aseptic technique. The sample centrifugation was done, and the serum was aliquot and stored in eppendorf tubes at −20°C for subsequent assays of IL-15 and TNF-*α*.

The concentration of both IL-15 and TNF-*α* were assayed using an ELISA kit from MyBioSource® (USA) according to the leaflet instructions provided.

According to the standard curve (one for IL-15 and other for TNF-*α*) that was generated by plotting the concentration of the standard against the optical density of each standard concentration, the serum concentrations of both IL-15 and TNF-*α* (for both patients and controls) were determined.

### 2.1. Ethical Considerations and Official Approvals

A patient agreement was obtained before data collection, and information was unnamed by replacing the name with a code and saved on a secured laptop to be used for research purposes only.

Administrative approvals were granted from the scientific committee of Al Kindy-College of Medicine-University of Baghdad on 30^th^ January 2022 with the approval code: 190.

### 2.2. Statistical Analysis

The data analysis was done by using the Statistical Package for Social Sciences (SPSS) version 26. A mean, standard deviation, and ranges were used for data presentation. Categorical data were presented as frequencies and percentages, and chi-squared test used for comparison between the data. If the frequency was <5, Fisher's exact test can be used. For comparison between continuous variables, independent *t*-test and analysis of variance (ANOVA) (two-tailed) were used.

*P* value was considered significant if it was <0.05.

## 3. Results

The whole number of participants was 60 and 38 patients were considered as a case group with AA, with 22 individuals without AA were considered as a control group.

The patients' ages were between 3–64 years for all participants with a mean and standard deviation (SD) of 24.9 years and ±13.9 years, respectively. The highest percentage of age in the case group was aged <20 years (47.4%) while for the controls, it was between 20–39 years (63.6%).

Regarding gender and age comparison between the case and the control group, a nonsignificant difference was observed (Supplementary Table 1)

The distribution of the case group by clinical characteristics are shown in Table 1. Namely, 39.5% of patients had the disease for <6 months, family history was positive in 21.1%, and the previous history was recorded in 28.9%.

Other body involvement was noticed in 7.9% of patients, exclamation marks were presented in 63.2%, and the pull test was positive in 71.1%, and nail changes were detected in 23.7%.

The most common form of AA was multilocularis (no. = 24) (63.2%), followed by unilocularis (no. = 8) (21.1%), and the least common were totalis and universalis (no. = 3) (7.9% each).

A comparison of the biomarkers between the two groups as shown in Table 2, revealed that IL-15 and TNF-*α* serum concentrations were higher significantly for case than the control group (2.35 ± 6.0) versus 0.35 ± 0.07 pg/mL, *P*=0.048; and 50.11 ± 60.77 versus 20.92 ± 28.0 pg/mL, *P*=0.014, respectively).

In the case group, the IL-15 level fell within 0.3–10.7 while the TNF-*α* level it was between 19–379.8. For the control, these levels fell between 0.3–0.6 and 3.9–138 for IL-15 and TNF-*α*, respectively.

For comparison between biomarkers and disease characteristics in case groups as in Table 3, no statistical difference was significant in the mean IL-15 level (*P* ≥ 0.05) regarding all characteristics. On the other hand, the mean level for TNF-*α* in totalis-type AA was higher significantly than other types (147.3 pg/mL, *P*=0.021), while there were nonsignificant differences for the mean TNF-*α* level (*P* ≥ 0.05) regarding all other characteristics of the case group.

## 4. Discussion

Alopecia areata (AA) is considered a common cause of reversible hair loss. The exact etiology is unknown, although many hypotheses suggest an association between the lymphocytic infiltration of the hair follicle and the disruption of the hair cycle due to a combination of multiple factors, including cytotoxic T-cell activity, cytokine release, and apoptosis [9].

Many cytokines participate significantly in diseases of the skin, particularly autoimmune skin diseases. For AA, the role of these cytokines is not well approved, although there is an association between AA and changes in the level of different cytokines [10].

Concurrently, many cytokines such as interleukin (IL)-7, IL-15, tumor necrosis factor-*α* (TNF-*α*), and interferon-*γ* (IFN-*γ*) are overexpressed in AA patients [11]. It is well known that lymphocytes development are enhanced by the action of interleukin-15 (IL-15) which has a role in certain diseases such as rheumatoid arthritis and multiple sclerosis that have an autoimmune etiology. IL-15 induces the synthesis of certain cytokines that participate in autoimmunity like, for example, TNF-*α* and IL-1*β*, by enhancing the maintenance of CD-8 memory T-cell through the inhibition of self-tolerance [12]. Keratinocytes of the epidermis san synthesize TNF-*α* which is an effective proliferation inhibitor. Additionally, a study showed that TNF-*α* causes a vacuolation in hair follicle, follicular melanocytes inactivation, and keratinization abnormalities for both the hair follicle bulb and inner root sheath [9].

In this current study, 60 patients were enrolled, comprising 38 patients who had AA (case group) and 22 healthy participants (control group).

In comparison for the level of both IL-15 and TNF-*α*, they were significantly higher in the case than in the control group (*P* < 0.05).

These results agreed with those of Ragab et al.'s study in 2020 [13]. Aşkın et al.'s study in 2021 [14] and Salem et al.'s study in 2019 [15] concluded that the level of IL-15 with AA were significantly higher in the case of control groups (*P* < 0.001).

For TNF-*α* serum level, this study agreed with the conclusions of Omar and colleagues in 2021 (*P* < 0.001) [16], Atwa and colleagues in 2016 (*P* < 0.05) [17], and Kasumagic–Halilovic and colleagues (*P*=0.044) [7].

In this study, there were no statistical differences in the mean levels of IL-15 (*P* ≥ 0.05) for all characteristics of the case group. These results agreed with those reported in Aşkın et al.'s study in 2021, which concluded that no statistical difference was found between males and females (*P* < 0.178) nor duration or severity of the disease (*P* > 0.05) regarding IL-15 serum levels for patient and control groups [14].

In the same way, Ragab et al.'s in their study in 2020 reported a similar finding, where the relation of IL-15 to patients' age and gender was assessed. They observed that there was no association between serum IL-15 and patients' gender. Even though autoimmune diseases are generally related to the gender of patients, no gender ascendancy is seen in AA (*P*=0.9). Likewise, it has been shown that there was no association between IL-15 level and patients age, (*P*=0.14), recurrence of disease, or history of AA in the same family of the patient (*P* > 0.05) [13].

Different findings were reported in a study by Salem et al. in 2019, in which among the case groups, they observed that serum level for IL-15 was higher for those with totalis type in comparison to patient with one or two patches and significantly higher in those with both scalp and body involvement compared to those with either scalp or body involvement (*P* < 0.05), while IL-15 levels between case groups were not related to age, gender, recurrence, or family history (*P* > 0.05) [15].

The differences reported above may be related to different sample sizes, the presence of other autoimmune conditions, and the duration and stage of the disease, which all affect the level of IL-15.

IL-15 trans-presentation can be blocked by targeting the cytokine receptor subunit IL-2/IL-15R*β*, and this is achieved through the use of Hu-Mik-*β*1 monoclonal antibody which is used now in clinical trials for treating those with autoimmune diseases [18, 19].

Regarding the mean TNF-*α* level in the present study, those with totalis-type had significantly higher mean TNF-*α* level than those of other types (*P* = 0.021). No statistically differences in the mean TNF-*α* levels were found when comparing all other characteristics (*P* ≥ 0.05).

These results agree with those of the Omar et al. study, in which there was no statistically significant difference in the serum levels of TNF-*α* in adults and children with AA (*P*=0.857). Moreover, there is no significant correlation in serum levels and severity (*P*=0.115). Patients with alopecia totalis/universalis have a higher serum concentration than in those with alopecia of patchy type, but without any significant correlation (*P*=0.39). Regarding the activity of the disease, there were no statistically significant differences in the serum cytokine level in patients with the active disease (56, 77.8%) compared with those with the inactive disease (16, 22.2%) (*P*=0.097) [16].

Moreover, in comparison between duration and severity of disease, the results agreed with those of Kasumagic–Halilovic et al., who reported that TNF-*α* levels between patients when considering the duration and severity of the disease were insignificant, even in comparison between those with localized and extensive disease (*P*=0.2272). Furthermore, those with a long disease duration had a high concentration of TNF-*α*, but without significant association (*P* > 0.05) [7]. Other studies found different conclusions. Atwa et al.'s study showed a significant correlation between TNF-*α* level and severity of disease (*r* = 0.247, *P*=0.031). No statistical significance had been found in the mean TNF-*α* concentration in comparison between those with different clinical types and those who had AA with or without atopy (*P* > 0.05) [17].

The differences reported among the above studies may be related to their different study designs as well as to disease severity, duration, comorbid conditions, and the presence of other autoimmune diseases.

It had been hypothesized that similar TNF-*α* levels in those with different types of AA give a clue toward the lack of immune reactions, while decreased TNF-*α* levels in the mild type may indicate a tendency for immunodeficiency in those with severe disease types [20]. Other studies found that serum levels of type-I TNF-*α* receptor were raised in those with the disease in comparison to nonaffected individuals. These finding propose that T-cells and keratinocytes activation are characteristic immune mechanisms in AA [21]. The participation of TNF-*α* in AA is through *e* elicitation of the catagen phase, as well as the breakdown of the keratinization of hair follicle [22].

Recent studies found a protective role for TNF-*α* in AA through the inhibition of plasmacytoid dendritic cell synthesis and IFN-*α* production and via prevention of the presentation of human leukocyte antigen.

This finding explains why TNF-*α* inhibitors may fail to cure patients with AA and may even be able to develop new cases in genetically predisposed individuals [22].

In conclusion, both IL-15 and TNF-*α* are markers for alopecia areata. The level for these biomarkers was not affected by duration or disease activity but it was affected by the types of disease as the concentration of IL-15 and TNF-*α* were elevated in those patients with totalis type than in other types of alopecia.

It is recommended to do a study in which the concentrations for both TNF- and IL-15 in patients with AA are measured at a basal level and at the time after the patient receives treatments.

## Figures and Tables

**Table 1:** Distribution of case groups by clinical characteristics.

Variables	Numbers (*n* = 38)	Percentage (%)
Duration of disease (months)		
<6	15	39.5
6–12	13	34.2
>12	10	26.3
Family history		
Positive	8	21.1
Negative	30	78.9
Previous history		
Positive	11	28.9
Negative	27	71.1
Other body area involvement		
Yes	3	7.9
No	35	92.1
Exclamation mark		
Yes	24	63.2
No	14	36.8
Pull test		
Positive	27	71.1
Negative	11	28.9
Nail change		
Yes	9	23.7
No	29	76.3

**Table 2:** Biomarkers comparison in studied groups.

Biomarkers (pg/ml)	Studied groups		*P* value
	Case Mean ± SD	Control Mean ± SD	
IL-15	2.35 ± 6.0	0.35 ± 0.07	0.048
TNF-*α*	50.11 ± 60.77	20.92 ± 28.0	0.014

IL-15 = interleukin-15, TNF-*α* = tumor necrosis factor alpha. SD = standard deviation. Pg/ml = picogram/ml.

**Table 3:** Mean IL-15 and TNF-*α* levels according to case group characteristics.

Variables	IL-15 (pg/mL) mean ± SD	*P* value	TNF-*α* (pg/mL) mean ± SD	*P* value
Age (years)				
<20	0.94 ± 0.6	0.069	68.47 ± 83.4	0.202
20–39	2.19 ± 2.5		36.18 ± 21.5	
≥40	7.9 ± 16.3		25.8 ± 6.5	
Gender				
Male	1.68 ± 2.4	0.507	39.07 ± 22.5	0.277
Female	2.95 ± 8.0		60.04 ± 80.7	
Duration of disease (months)				
<6	1.9 ± 2.5	0.326	38.64 ± 25.5	0.568
6–12	1.03 ± 0.78		51.56 ± 32.7	
>12	4.75 ± 11.4		65.43 ± 110.7	
Family history				
Positive	0.75 ± 0.39	0.111	48.91 ± 18.1	0.914
Negative	2.78 ± 6.73		50.43 ± 68.1	
Previous history				
Positive	1.7 ± 2.9	0.577	44.21 ± 28.2	0.607
Negative	2.61 ± 6.9		52.51 ± 70.2	
Other body area involvement				
Yes	0.66 ± 0.05	0.093	29.9 ± 5.4	0.056
No	2.49 ± 6.3		51.84 ± 63.7	
Exclamation mark				
Yes	1.65 ± 2.1	0.478	60.31 ± 73.9	0.092
No	3.55 ± 9.6		32.62 ± 18.5	
Pull test				
Positive	1.57 ± 2.0	0.432	56.54 ± 70.3	0.145
Negative	4.27 ± 10.9		34.33 ± 20.7	
Nail change				
Yes	0.65 ± 0.11	0.091	48.51 ± 27.6	0.894
No	2.87 ± 6.8		50.61 ± 68.3	
Types of alopecia areata				
Multilocularis	1.68 ± 2.1	0.458	47.7 ± 30.7	0.021
Unilocularis	5.4 ± 12.8		27.57 ± 11.0	
Universalis	1.3 ± 1.0		32.23 ± 8.2	
Totalis	1.6 ± 0		147.3 ± 200.6	

## Data Availability

The data and materials related to the present work are included within this article.

## Supplementary Materials

Supplementary Materials Supplementary Table 1: Comparison between studied groups by age and gender.
