# Supplementary material for: Interleukin-15 and Tumor Necrosis Factor-α in Iraqi Patients with Alopecia Areata
Source: Dermatol Res Pract. 2023 May 10;2023:5109772. doi: 10.1155/2023/5109772 (PMC10191747; doi:10.1155/2023/5109772)
Supplement: Supplementary Materials — Supplementary Table 1: Comparison between studied groups by age and gender. [file 5109772.f1.docx]

**Table 1: Comparison between studied groups by age and gender** Supplementary

| General Characteristics | Studied Group | | P-Value |
| --- | --- | --- | --- |
|  | **Case (%)**  **n= 38** | **Control (%)**  **n= 22** |  |
| Gender | | | |
| Male | 18 (47.4) | 10 (45.5) | **0.886** |
| Female | 20 (52.6) | 12 (54.5) |  |
| Mean ± SD           Mean ± SD | | | |
| Age (Years) | 22.63±15.3 | 28.81±10.1 | **0.065** |
